# Case report of *Enterococcus thailandicus* bacteremia, with genomic characterization of its clinical isolate

**DOI:** 10.1128/asmcr.00104-25

**Published:** 2025-12-08

**Authors:** Yusuke Tsuda, Taro Noguchi, Shuji Yamamoto, Koh Shinohara, Yasuhiro Tsuchido, Masaki Yamamoto, Yasufumi Matsumura, Miki Nagao

**Affiliations:** 1 Department of Clinical Laboratory Medicine, Kyoto University Graduate School of Medicine 593288 , Kyoto City, Kyoto, Japan; 2 Department of Infection Control and Prevention, Kyoto University Hospital 34797, Kyoto City, Kyoto, Japan; 3 Department of Gastroenterology, Kobe City Nishi-Kobe Medical Center 34797, Kobe City, Hyogo, Japan; Pattern Bioscience, Austin, Texas, USA

**Keywords:** phylogenetic analysis, bacteremia, *Enterococcus thailandicus*

## Abstract

**Background:**

*Enterococcus thailandicus* was initially isolated from food in Thailand, and a single clinical case involving an intra-abdominal abscess was reported. No reports on the bacteremia or whole-genome sequencing of clinically isolated *E. thailandicus* have been published.

**Case Summary:**

A 50-year-old woman with intestinal Crohn’s disease was admitted because of bloody stools. She had been treated twice previously for a perianal abscess. She developed a fever on day 15 of hospitalization. Both blood and urine cultures were polymicrobial and included *E. thailandicus*, which was susceptible to penicillins. She was treated for the perianal abscess and pyelonephritis with intravenous penicillins combined with beta-lactamase inhibitors for 2 weeks, followed by oral amoxicillin-clavulanic acid for 4 weeks. The identities of the isolates in the blood and urine cultures were confirmed using whole-genome analysis. No antimicrobial resistance genes, virulence genes, or plasmid replicons were identified in these isolates. Phylogenetic analysis of the clinical isolates and 21 *E. thailandicus* isolates in public databases indicated a relatively close lineage to that obtained from pig stool samples in Japan. No antimicrobial resistance genes conferring resistance to penicillins were identified in any of the *E. thailandicus* genomes.

**Conclusion:**

Phylogenetic analysis suggested that infection with *E. thailandicus* in this patient may have originated in Japan. The prognosis after treatment with penicillins was favorable.

## INTRODUCTION

*Enterococcus thailandicus* was isolated from food in Thailand (1). It is a senior subjective synonym of *Enterococcus sanguinicola*, of which two clinical isolates were recovered from human blood in 2004 and proposed as a new species in October 2008 (2, 3). However, *E. thailandicus* was isolated in July 2008, and it was demonstrated that *E. sanguinicola* and *E. thailandicus* represent the same species, based on the identity of their 16S rRNA and *rpoB* gene sequences in 2011 (4). The clinical courses of the patients from whom *E. sanguinicola* was isolated were not described.

Phylogenetic analysis of the 16S rRNA and *rpoA* genes revealed that *E. thailandicus* is relatively close to *Enterococcus durans*, *Enterococcus faecium*, and *Enterococcus hirae* (1). The first clinical report of an intra-abdominal abscess caused by *E. thailandicus* was recently documented (5). However, no other reports of bloodstream infections caused by *E. thailandicus*, or genomic analyses of clinical isolates have been published. Herein, we present a case of *E. thailandicus* bacteremia and describe microbiological features using whole-genome sequencing (WGS).

## CASE PRESENTATION

A 50-year-old woman was admitted to our hospital with bloody stools. She had a history of intestinal Crohn’s disease and was treated with ustekinumab and an elemental diet (ELENTAL︎) until admission. She had been treated with flomoxef for perianal abscesses 6 and 8 years prior to this admission. Although the abscess cavity persisted, no clinical deterioration was observed in the absence of antimicrobial treatment. The patient was a full-time homemaker. She had not traveled abroad in the past 10 years and had no regular contact with animals. Fasting, initiation of infliximab at 5 mg/kg, and argon plasma coagulation via small bowel endoscopy resolved the bloody stools by hospital day 7.

On hospital day 15, she developed fever, stomach irritation, and diarrhea. Physical examination revealed no tenderness in the stomach or costovertebral angle. Blood tests revealed a white blood cell count of 6,510 cells/µL (reference range [RR]: 3,300–8,600), a C-reactive protein level of 4.1 mg/L (RR: ≤1.4), and a procalcitonin level of 0.02 µg/L (RR: <0.05). Urinalysis performed the day after the initiation of piperacillin-tazobactam at 4.5 g every 8 hours revealed one to four leukocytes per high-power field and a bacterial count of 30.3/µL. Computed tomography (CT) imaging showed reduced perianal abscess compared with the CT findings on admission, with no abnormalities in the urinary tract. Two sets of blood cultures and urine culture were subsequently obtained, and she was administered piperacillin-tazobactam.

On the following day, both sets of blood cultures (BD BACTEC; Becton, Dickinson and Company, New Jersey, USA) became positive. Gram-negative bacilli were identified in one set, whereas gram-positive cocci were identified in the other. *Escherichia coli*, *E. thailandicus*, *Enterococcus avium*, and *Enterococcus gallinarum* were identified using a Bruker MALDI Biotyper︎ (Bruker, Billerica, USA; Reference Library v.6.0.0). The confidence score of *E. thailandicus* for MALDI identification was 2.02. Antimicrobial susceptibility testing using MicroScan WalkAway (Beckmann-Coulter, Germany) showed susceptibility of *E. thailandicus* and *E. coli* to penicillins (Table 1). Urine cultures revealed the presence of *E. thailandicus*, *E. coli*, and *Corynebacterium amycolatum*. As a stool culture was not obtained, the carriage of *E. thailandicus* was unknown. Considering her abdominal symptoms, fever, and isolation of the same bacteria from both blood and urine cultures, the patient was diagnosed with a perianal abscess and pyelonephritis. Based on the antimicrobial susceptibility testing, she received piperacillin-tazobactam for 8 days and ampicillin-sulbactam at 3 g every 8 hours for 6 days. At discharge, she was prescribed amoxicillin-clavulanic acid at 375 mg/125 mg every 8 hours for the perianal abscess. At a 1-month follow-up, she remained well without relapse.

**TABLE 1:** Antimicrobial susceptibility tests of the isolates *Enterococcus thailandicus* KUN2022-1874 and KUN2022-1934^*a*^

Antimicrobial agent	KUN2022-1874		KUN2022-1934	
	MIC (μg/mL)	Interpretation	MIC (μg/mL)	Interpretation
Penicillin	4	S	4	S
Ampicillin	≤2	S	≤2	S
Levofloxacin	2		2	S
Minocycline	>8	R	>8	R
Vancomycin	≤0.5	S	≤0.5	S
Daptomycin	1	S	1	S
Linezolid	2	S	2	S
Rifampicin	>2	R	>2	R

^*a*^ MIC, minimal inhibitory concentration; R, resistant; S, susceptible. Antimicrobial susceptibility testing was conducted using MicroScan WalkAway. Interpretations were decided according to the Clinical and Laboratory Standards Institute M100 ED35.

### Microbiological and genomic characteristics of the clinical isolates

**Fig 1:**
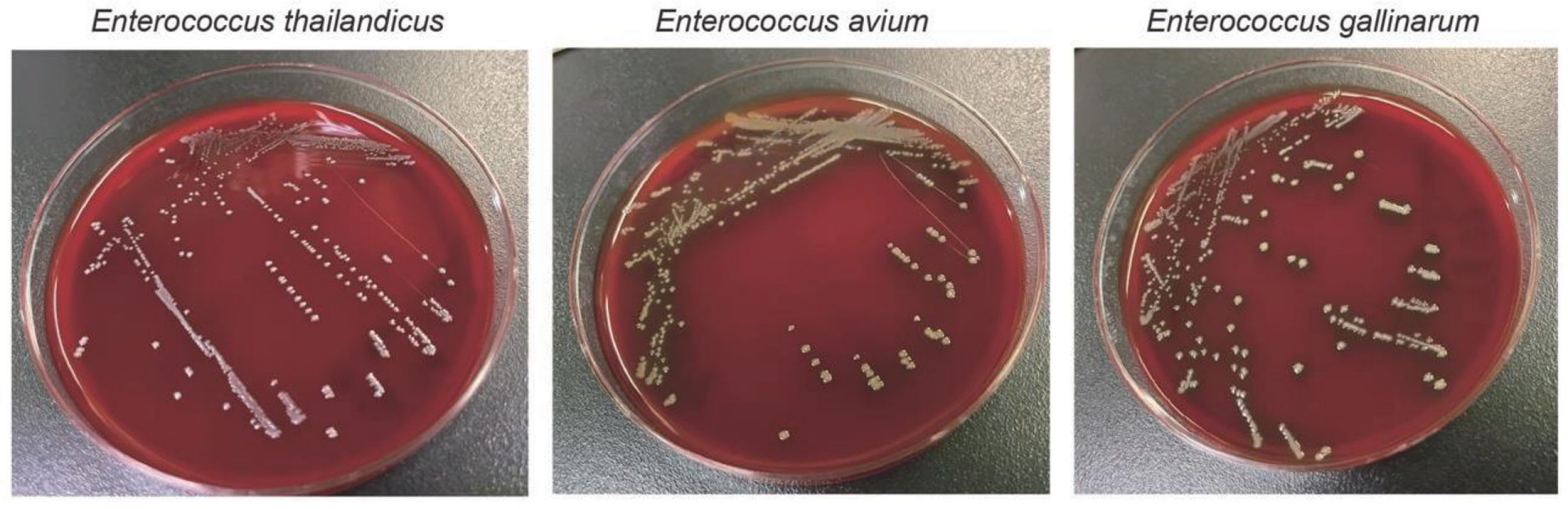
Colonies of enterococci. Clinical isolates of *Enterococcus thailandicus* KUN2022-1874, *Enterococcus avium*, and *Enterococcus gallinurum* from blood culture were incubated on Columbia blood agar at 35℃ for 24 hours. Colonies of *E. thailandicus* appear white, small, and non-hemolytic, allowing differentiation from other enterococci.

Colonies of *E. thailandicus* appeared white, small, and non-hemolytic (Fig. 1). Using API︎20 STREP (bioMérieux, Marcy l’Etoile, France), biochemical characterization of the two clinical isolates—KUN2022-1874 from blood culture and KUN2022-1934 from urine culture—revealed identical profiles (Table 2). Both isolates were positive for PYRase activity, LAPase activity, and hydrolysis of esculin and arginine. Acid production was observed from D-ribose, D-mannitol, D-lactose, and D-trehalose.

**TABLE 2:** Reaction of the isolates *Enterococcus thailandicus* KUN2022-1874 and KUN2022-1934 in API︎20 STREP^*a*^

Isolate	ATCC 35667 *E. faecium*	KUN2022-1874 *E. thailandicus*	KUN2022-1934 *E. thailandicus*
VP	+	+	+
HIP	+	−	−
ESC	+	+	+
PYRA	+	+	+
αGAL	−	−	−
βGUR	−	−	−
βGAL	+	−	−
PAL	−	−	−
LAP	−	+	+
ADH	+	+	+
RIB	+	+	+
ARA	+	−	−
MAN	+	+	+
SOR	−	−	−
LAC	+	+	+
TRE	+	+	+
INU	−	−	−
RAF	−	−	−
AMD	+	−	−
GLYG	−	−	−

^*a*^ ADH, L-arginine; AMD, starch; ARA, L-arabinose; αGAL, 6-bromo-2-naphthyl-αD-galactopyranoside; βGAL, 2-naphthyl-ßD-galactopyranoside; βGUR, naphthol ASBI-glucuronic acid; ESC, esculin ferric citrate; GLYG, glycogen; HIP, hippuric acid; INU, inulin; LAC, D-lactose; LAP, L-leucine-ß-naphthylamide; MAN, D-mannitol; PAL, 2-naphthyl phosphate; PYRA, pyroglutamic acid-ß-naphthylamide; RIB, D-ribose; RAF, D-raffinose; SOR, D-sorbitol; TRE, D-trehalose; VP, sodium pyruvate.

Genomic DNA from the two isolates was extracted using MagNA Pure 96 system (Roche, Basel, Switzerland). Libraries of KUN2022-1974 and KUN2022-1934 were prepared using an Illumina DNA Prep kit (Illumina, San Diego, USA) and sequenced on an Illumina NextSeq 1000 instrument (151 and 300 bp paired-end reads, respectively). Reads were assembled using SPAdes v.3.15.5. Evaluation using QUAST v.5.0.2 and bamcov v.0.1.1 showed that the draft genome of KUN2022-1874 and KUN2022-1934 had a total length of 2,630,973 and 2,461,271 bp with 36.69% and 36.72% guanine-cytosine content and consisted of 97 and 37 contigs with an average coverage of 150× and 74.7×, respectively (Table 3) (6, 7). Using the OrthoANIu algorithm, a markedly average nucleotide identity (99.4%) and coverage (70.3%) between the KUN2022-1874 and reference sequence (accession number CP023074.1) confirmed its identification as *E. thailandicus* (8). Furthermore, variant calling with Snippy v.4.6.0 identified two nucleotide variants between KUN2022-1873 and KUN2022-1934, supporting that the two isolates were nearly identical (9). Virulence genes, such as *cyl* and *hyl*, were not identified using VirulenceFinder v.3.0.0 (10, 11). BLASTP analysis using six penicillin-binding proteins (PBPs) from *E. faecium* (accession number CP038996.1) revealed that all of the PBPs were identified in KUN2022-1874 and KUN2022-1934. The identity of PBP4 in KUN2022-1874 was 73.2% compared to that of *E. faecium*. No antimicrobial resistance genes and replicon sequences were detected using ResFinder v.4.2.3 and PlasmidFinder v.2.1.6 (11–14)

**TABLE 3:** Assembly statistics of the isolates *Enterococcus thailandicus* KUN2022-1874 and KUN2022-1934

Statistic	KUN2022-1874	KUN2022-1934
Total length (bp)	2,630,973	2,461,271
N50 (bp)	105,219	377,306
N75 (bp)	55,490	149,910
L50	9	3
L75	17	6
No. of N’s per 100 kbp	0	0
No. of contigs	97	37
Largest contig (bp)	245,394	723,332
GC%	36.69	36.72

### Phylogenetic analysis of *E. thailandicus* isolates

**Fig 2:**
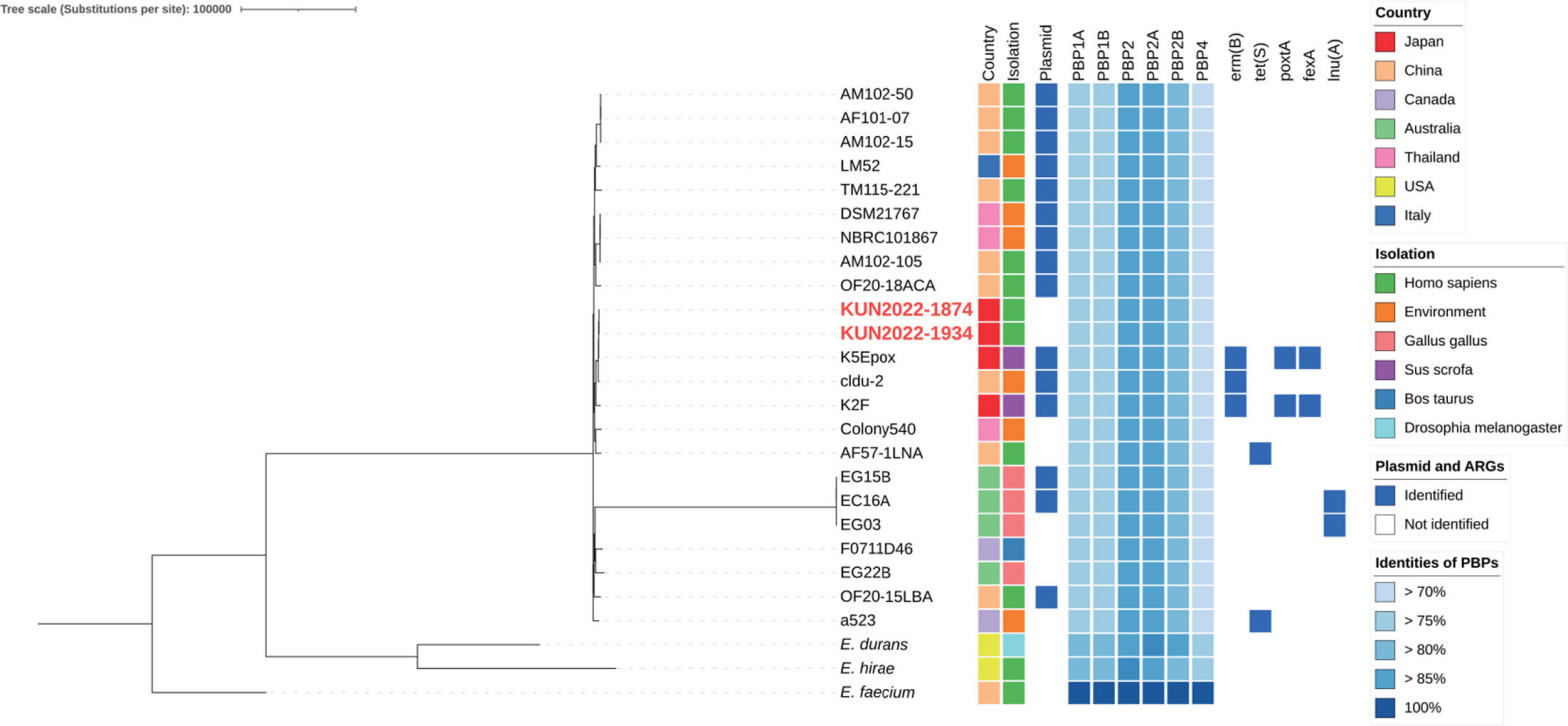
Phylogenetic tree of *Enterococcus thailandicus*. A total of 21 data sets of *Enterococcus thailandicus* from the NCBI database, *Enterococcus durans* (CP022930.1), *Enterococcus faecium* (CP038996.1), *Enterococcus hirae* (CP023011.2), and the clinical isolates KUN2022-1874 and KUN2022-1934 were included in the phylogenetic analysis. The phylogenetic tree was visualized using the Interactive Tree Of Life web server (17). Antimicrobial resistance genes (ARGs) which were detected in more than two strains were presented. Locus tags of *pbp* genes were *pbp1A*, E6A31_06625; *pbp1B*, E6A31_09430; *pbp2*, E6A31_03785; *pbp2A*, E6A31_12780; *pbp2B*, E6A31_04730; and *pbp4*, E6A31_07265.

Phylogenetic analysis of 21 WGS data sets of *E. thailandicus* registered in the National Center for Biotechnology Information (NCBI) in August 2023 and the two clinical isolates was conducted using Snippy, Gubbins v.3.3.1, and IQ-TREE v.2.3.0 (Fig. 2 and Table 4) (9, 15, 16). *E. thailandicus* has been isolated from samples in East Asia, Australia, Canada, and Italy. Of the reported isolates, eight (38.0%) and seven (33.3%) isolates were obtained from human and animals, respectively. Phylogenetic analysis revealed that KUN2022-1874 and KUN2022-1934 clustered on the same branch as K5Epox and cldu-2 and were closely related to K5Epox, which was isolated from the pig stool samples in Japan (17). BLASTP analysis revealed that all isolates from NCBI harbored six PBPs . Genes conferring resistance to macrolide, lincosamide, tetracycline, and chloramphenicol were identified in seven isolates. Plasmid replicon sequences were identified in 15 of the 21 isolates (71.4%) (11, 12).

**TABLE 4:** Details of 21 *E. thailandicus* strains registered in NCBI

Strain	Host	Isolation source	Country	Collection yr	Accession no.
F0711D46	*Bos taurus*	Feces	Canada	2005	GCF_001652875.1
LM52	Environment	–^*b*^	Italy	2012	GCF_001495195.1
a523	Environment	Raw sewer	Canada	2013	GCF_002290025.1
AF57-1LNA	*Homo sapiens*	Fecal material	China	2014	GCF_027681785.1
AF101-07	*Homo sapiens*	Fecal material	China	2014	GCF_027687245.1
AM102-15	*Homo sapiens*	Fecal material	China	2014	GCF_027660045.1
AM102-50	*Homo sapiens*	Fecal material	China	2014	GCF_027659885.1
AM102-105	*Homo sapiens*	Fecal material	China	2014	GCF_027660215.1
OF20-15LBA	*Homo sapiens*	Fecal material	China	2014	GCF_027693865.1
OF20-18ACA	*Homo sapiens*	Fecal material	China	2014	GCF_027693845.1
TM115-221	*Homo sapiens*	Fecal material	China	2014	GCF_027678665.1
EC16A	*Gallus gallus*	–	Australia	2016	GCF_027859335.1
EG03	*Gallus gallus*	–	Australia	2016	GCF_027859305.1
EG15B	*Gallus gallus*	–	Australia	2016	GCF_027859295.1
EG22B	*Gallus gallus*	–	Australia	2016	GCF_027859355.1
DSM21767	Environment	Fermented sausage	Thailand	2016	GCF_001886265.1
cldu-2	Environment	Dust from chicken farm	China	2019	GCF_030179535.1
NBRC101867	Environment	Mum (fermented sausage)	Thailand	2019^*a*^	GCF_007989705.1
K2F	*Sus scrofa*	Excrement	Japan	2021	GCF_030270205.1
K5Epox	*Sus scrofa*	Excrement	Japan	2021	GCF_030270265.1
Colony540	Environment	Food	Thailand	2021^*a*^	GCF_019265385.1

^*a*^ The year of registration in the NCBI database as collection year was not indicated.

^*b*^ –, data not available.

## DISCUSSION

In this report, we present a case of *E. thailandicus* bacteremia in a 50-year-old woman and describe the microbiological features of its clinical isolates using WGS. Polymicrobial bloodstream infections are most commonly associated with intra-abdominal infections; however, urinary tract infections are also reported in approximately 11% of patients (18). In this case, the isolates from the blood and urine cultures were nearly identical, and pyelonephritis was also determined as the cause of bacteremia. Similarly, *E. thailandicus* has previously been isolated from a polymicrobial intra-abdominal infection (5). Enterococci are known to facilitate polymicrobial infections, suggesting that *E. thailandicus* may be associated with such infections (19).

*E. thailandicus* FP48-3 and TC1 and *E. sanguinicola* BAA-781 and CCUG 47884 hydrolyzed esculin and produced acid from ribose and mannitol (1, 2, 4, 20). However, TC1, BAA-781, and CCUG 47884 produced acid from trehalose, while FP48-3 did not, indicating that the profiles of our clinical isolates matched those of TC1, ATCC BAA-781, and CCUG 47884. As the whole-genome sequences of *E. sanguinicola* are not available in the NCBI database, we were unable to perform a comparative genomic analysis with our isolates. Because our clinical isolates were confirmed to be *E. thailandicus* using average nucleotide identity, we consider this to be the first documented clinical case of *E. thailandicus* bacteremia.

Subsequent isolations of *E. thailandicus* have been reported in sewage and animals (21, 22). Although isolates from human stool samples have been reported via metagenome analysis, they have not been characterized (23). Because the current clinical isolates were closely related to the strain in Japan, and the patient had no history of international travel, the infection likely occurred in Japan.

In enterococci, aminoglycosides, cephalosporins, clindamycin, and trimethoprim-sulfamethoxazole are not effective clinically. Penicillin resistance in *E. faecium* has been linked to the *pbp4(5*) gene (24). A clinical isolate formerly identified as *E. sanguinicola,* reported in 2004 (ATCC BAA-781), carried the *vanA* gene, which confers resistance to vancomycin (2). In contrast, *E. thailandicus* isolates from animals were typically susceptible to penicillins (13, 20, 25). Notably, the strain K5Epox, closely related to our clinical isolates, was non-susceptible to linezolid due to the presence of the *poxtA* gene (26). The clinical isolate of *E. thailandicus* from intra-abdominal infection was susceptible to ampicillin, vancomycin, and linezolid, and the patient was treated with meropenem, levofloxacin, and vancomycin (5). No reports of penicillin-resistant *E. thaliandicus* have been published since 2005. Therefore, the low identity of PBP4(5) in *E. faecium* and other antimicrobial resistance genes may support the use of penicillins for the empirical therapy.

In conclusion, infections caused by *E. thailandicus* may be treated effectively with penicillins. Additional clinical reports and genomic analyses are essential to clarify its microbiological characteristics and reinforce clinical management.

## Data Availability

Raw read sequences of KUN2022-1874 and KUN2022-1934 were registered in the NCBI SRA under accession numbers DRX665332 and DRX764865, respectively.
